# The Role of Gut Microbiota Modification in Nonalcoholic Fatty Liver Disease Treatment Strategies

**DOI:** 10.1155/2024/4183880

**Published:** 2024-10-16

**Authors:** Hessam Yaghmaei, Amirhossein Bahanesteh, Masood Soltanipur, Sobhan Takaloo, Mahdi Rezaei, Seyed Davar Siadat

**Affiliations:** ^1^Department of Biology, Science and Research Branch, Islamic Azad University, Tehran, Iran; ^2^Department of Mycobacteriology and Pulmonary Research, Pasteur Institute of Iran, Tehran, Iran; ^3^Student Research Committee, Faculty of Medicine, Shahed University, Tehran, Iran; ^4^Cardiovascular Research Center, Shahid Beheshti University of Medical Sciences, Tehran, Iran; ^5^Biomedical Engineering Department, Hamedan University of Technology, Hamedan, Iran; ^6^Microbiology Research Center (MRC), Pasteur Institute of Iran, Tehran, Iran

**Keywords:** gut microbiota modification, microbiome dysbiosis, nonalcoholic fatty liver disease (NAFLD), prebiotics and probiotics, treatment strategies

## Abstract

One of the most common chronic liver diseases is nonalcoholic fatty liver disease (NAFLD), which affects many people around the world. Gut microbiota (GM) dysbiosis seems to be an influential factor in the pathophysiology of NAFLD because changes in GM lead to fundamental changes in host metabolism. Therefore, the study of the effect of dysbiosis on the pathogenicity of NAFLD is important. European clinical guidelines state that the best advice for people with NAFLD is to lose weight and improve their lifestyle, but only 40% of people can achieve this goal. Accordingly, it is necessary to provide new treatment approaches for prevention and treatment. In addition to dietary interventions and lifestyle modifications, GM modification-based therapies are of interest. These therapies include probiotics, synbiotics, fecal microbiota transplantation (FMT), and next-generation probiotics. All of these treatments have had promising results in animal studies, and it can be imagined that acceptable results will be obtained in human studies as well. However, further investigations are required to generalize the outcomes of animal studies to humans.

## 1. Introduction

Nonalcoholic fatty liver disease (NAFLD) is a growing chronic liver disease worldwide [1]. This disease is associated with an increased incidence of metabolic diseases such as insulin resistance (IR), obesity, and Type 2 diabetes mellitus (T2DM) [2]. Saturated fatty acid–rich diet, sugar-rich drinks, refined carbohydrates, fructose, western diets, and imbalance in calorie intake and consumption can lead to obesity and NAFLD, which indicates that the incidence of this disease strongly depends on lifestyle and dietary patterns [3]. Various factors are involved in the pathophysiology of NAFLD such as IR, inflammation, de novo lipogenesis (DNL), and oxidative stress, which adds to the complexity of understanding the disease [4].

Another factor involved in the pathophysiology of NAFLD is the gut microbiota (GM). Its role has been studied in numerous ways such as fecal microbiota transplantation (FMT), diet habits and lifestyle, weight loss, probiotics, and pharmacological approaches. The FMT method shows that the obesity or T2DM phenotype is transmitted from mice into recipient mice by FMT [5]. Long-term diet habits are an influential factor in shaping the composition and structure of the GM community, and diet modification is equally effective in it [6], which suggests that diet can affect the GM and explain the possible link between NAFLD and an unhealthy diet. Accordingly, it has been shown that weight loss through dietary interventions is associated with quantitative and qualitative alterations in GM, which modulates the GM [7].

The study of GM alterations provides a better understanding of the clinical approach to NAFLD treatment. Hence, research has focused on providing new treatment strategies based on the modification of GM to improve NAFLD or reduce the progression of this disease. This article reviews these strategies for modulating the GM and describes how these therapeutic interventions affect the gut–liver axis.

## 2. NAFLD Treatment Methods Based on the Modification of GM

### 2.1. Probiotics

Probiotics are living microorganisms that if administered adequately can contribute to the health of the host [8]. Probiotic genera such as *Lactobacillus* and *Bifidobacterium* are alternately used to treat NAFLD/NASH [9]. Most probiotic products on the market are made of *Lactobacillus*, and it seems that the best probiotic supplement for animal models is the genus *Lactobacillus* [10]. Probiotic microorganisms coexisting with the host can fortify the intestinal barrier, participate in immunomodulation and immunoregulation, and suppress pathogenic microbial agents [11]. Probiotics are considered beneficial and potentially therapeutic microorganisms, so they may have advantageous and preventive effects on NAFLD. Many animal studies have revealed that glycometabolism and lipometabolism are also affected by probiotics [12]. Lactobacilli can alter the GM and prevent the development and promotion of steatohepatitis. Decreased hepatic steatosis was observed in diet-induced obese rats after 8 weeks of oral administration of *Lactobacillus rhamnosus*, which was also confirmed by liver biopsy [13]. In mice with NAFLD due to a high-fructose diet, *Lactobacillus rhamnosus GG* was able to strengthen the intestinal barrier and improve cholesterol metabolism and disease-related symptoms [14]. Treatment with *Bifidobacterium* supplementation in NAFLD rats induced by a high-fat diet (HFD) reduced fat accumulation [15]. Also, supplements containing *Bifidobacterium* could decrease the body mass index (BMI) in people with NAFLD [16]. Studies show that administration of probiotics decreased aminotransferase levels [17, 18]. In particular, *Lactobacillus bulgaricus* and *Streptococcus thermophilus* significantly reduced alanine aminotransferase (ALT) levels for 3 months [18].

Also, administering a mixture of probiotics can improve systemic inflammation and normalize the pattern of fecal microbiota, suggesting that probiotics can be an interesting treatment option for NAFLD [19]. Administration of probiotic VSL #3, which includes *Streptococcus thermophilus*, *Bifidobacterium longum*, *Lactobacillus acidophilus*, *Lactobacillus plantarum*, and *Lactobacillus casei*, in animals with NAFLD indicates that the severity of tissue damage of the liver is improved and probiotic treatment reduces the activation of aminotransferases [20]. Another 3-month intervention with probiotic VSL #3 in adults revealed an improvement in liver enzymes, although this is an indirect marker of the NAFLD severity [21]. Also, this probiotic supplement can decrease the markers of lipid peroxidation, cyclooxygenase-2 (COX-2), inducible nitric oxide synthase (iNOS), and tumor necrosis factor-alpha (TNF-*α*) in mice fed an HFD [22]. In another animal study, liver histology and total free fatty acid (FFA) content and serum ALT levels were improved by treatment with a probiotic mixture (including *Lactobacillus*, *Streptococcus thermophilus*, and *Bifidobacterium*) [23]. Likewise, a decrease in inflammatory markers such as TNF-*α* and markers of oxidative stress and liver enzymes in individuals with NAFLD was demonstrated by treatment for 2–3 months with VSL #3 supplementation and synbiotic (a mixture of pro-/prebiotic) [21]. A clinical trial showed that the VSL #3 probiotic supplement effectively lowered BMI, raised glucagon-like peptide 1 (GLP-1) levels, and improved NASH and childhood obesity [24]. Treatment with the mixture of multispecies probiotics including *Lactobacillus rhamnosus*, *Lactobacillus acidophilus*, *Pediococcus pentosaceus*, *Lactobacillus paracasei*, *Bifidobacterium breve*, and *Bifidobacterium lactis* in a randomized double-blind controlled trial for 12 weeks showed a decrease in BMI, and the reduction in liver fat and triglyceride levels was significant [25].

The effects of 6-month treatment with a probiotic cocktail were evaluated by proton nuclear magnetic resonance (^1^H NMR) and showed a significant reduction in intrahepatic triglyceride content [26]. Probiotics were found to cause a small but meaningful reduction in liver fat compared with the placebo group [25]. Probiotic cocktails increase bacterial genera such as *Agathobaculum*, *Dorea*, *Blautia*, and *Ruminococcus*, associated with decreased steatosis. In an RCT study, capsules containing the probiotics *Lactobacillus*, *Enterococcus*, and *Bifidobacterium* were administered to NAFLD patients, and ultrasound measured a significant reduction in liver fat [27]. Treatment of children with a probiotic cocktail (e.g., VSL #3) for 4 months showed a significant improvement in ultrasound-measured hepatic steatosis compared with the placebo group [24].

In general, previous studies show heterogeneous results of probiotic cocktails' impact on NAFLD according to the length of treatment, the type of combination, and probiotic dosages. Despite variations in individual responses, the use of probiotic cocktails is not dangerous in the short term and may improve liver steatosis to some extent or at least prevent it from getting worse compared to a placebo. These beneficial combinations lead to dysbiosis correction, reduction in endotoxemia, strengthening of the intestinal barrier, reduction in BMI, and improved insulin sensitivity [16, 24, 25, 27]. Therefore, probiotic cocktails could potentially serve as valuable tools for preventing NAFLD progression. Nevertheless, future studies should explore ideal dosages, durations, and compositions to ensure maximum effectiveness.

**Figure 1:**
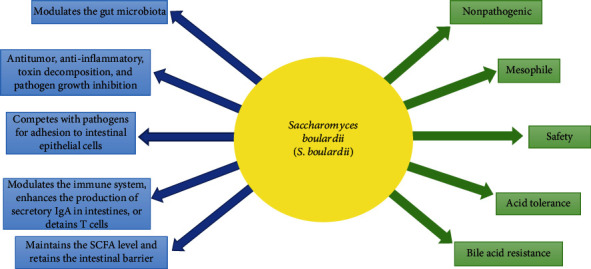
The characteristics (green arrows) and capabilities (blue arrows) of *Saccharomyces boulardii* (*S. boulardii*).

The *Saccharomyces boulardii* (*S. boulardii*) is considered a probiotic, and its use has shown hopeful results, especially when it is used in combination with other traditional probiotics [28]. This probiotic provides the environment for the growth of lactic acid bacteria and *Bifidobacterium* by consuming oxygen [28]. The *S. boulardii* has a good characteristic such as nonpathogenicity, mesophile, safety, acid tolerance, and bile acid resistance [29–31] (Figure 1). This yeast strain has varied remarkable features and abilities which are mentioned as follows (Figure 1): (1) As a probiotic, it can modulate the GM [32]. (2) It has antitumor and anti-inflammatory activity, aids in toxin decomposition, and inhibits pathogen growth [29]. (3) It tends to adhere to intestinal epithelial cells (IECs) and competes with pathogens for adhesion to IECs [33]. (4) It can modulate the immune system, inhibit inflammation, enhance the production of secretory IgA in intestines, or detain T cells [34, 35]. (5) It maintains the SCFA level and aids in retaining the intestinal barrier [36].

The probiotic *S. boulardii* is widely used in clinical practice, and it is effective in some studies. Seliverstov et al. showed that the use of lyophilized *S. boulardii* within 3 months in NAFLD patients can notably reduce the *Bacteroides fragilis* group and *Escherichia coli.*Additionally, in the treatment group, improved symptoms and quality of life, reduction in body weight, and significant decrease in VLDL level and atherogenic index were observed [37]. Liu, Li, and Wang demonstrated that the oral administration of *S. boulardii* in an animal model that was fed an HFD could lead to a notable decrease in weight, ALT and AST, endotoxin, TNF-*α*, and IFABP levels. Reduction of liver steatosis and changes in intestinal microbiota (remarkable increase in *Bacteroides* and significant decrease in *E. coli*) were witnessed [38]. In an animal study, oral gavage daily of *S. boulardii* for 4 weeks to obese mice reduced weight and fat mass and improved hepatic steatosis and inflammation. Also, *S. boulardii* increased cecal weight and altered the taxonomy of GM at various levels (*Firmicutes*, *Proteobacteria*, and *Tenericutes* decreased while *Bacteroidetes* increased) [39]. Another similar study showed that oral gavage of this probiotic at a dose of 7.5 × 10^9^CFU/kg per day for 8 weeks in mice with diet-induced steatohepatitis resulted in weight loss, inflammation, and improved hepatic steatosis and endotoxemia. Also, in treated rats, the ratio of *E. coli* to *Bacteroides* was adjusted, indicating interactions between fungi and bacteria [40]. Yang et al. found that oral administration of *S. boulardii* to an animal model can improve liver function and intestinal permeability and decrease steatosis and inflammation. In addition, it can suppress both hepatic inflammatory gene expression and fibrogenic gene expression. Also, it can significantly increase beneficial microbiota and microbiota diversity [41]. *S. Boulardii* can improve fecal butyrate levels in individuals with long-term total enteral nutrition [36]. Also, it can be effective in dyslipidemia treatment [42]. *S. boulardii* can regulate liver peptide levels that are responsible for the renin–angiotensin system in diabetes-induced liver injury mice [43]. This probiotic is generally safe, tolerable, and useful, but it should be noted that only a few cases of fungemia have been reported following *Clostridium difficile* colitis [44]. This emphasizes the importance of closely monitoring probiotic use and studying their effectiveness, especially in humans. Also, there is limited research on the impact of *S. boulardii* on liver diseases, highlighting the importance of giving more attention to this matter. Therefore, further studies should be conducted on the possible role of intestinal fungi in the pathogenesis of NAFLD.

Sarcopenia, the age-related loss of skeletal muscle mass and function, is increasingly recognized as a significant comorbidity in patients with NAFLD. Recent studies have highlighted the strong association between sarcopenia and NAFLD, emphasizing the need to understand their interrelationship for better management of both conditions. The systematic review by Li, Kim, and Ahmed demonstrates that sarcopenia is highly prevalent among NAFLD patients, with an increased rate in those with more severe NAFLD [45]. As highlighted in Tarantino et al.'s study, the role of the microbiome in sarcopenia is an important aspect that requires further investigation. GM dysbiosis can lead to the degradation of branched-chain amino acids in muscle and decrease the expression of insulin-like growth factor 1, which has a critical role in muscle growth and repair [46]. The GM is linked to the gut–brain–muscle axis, influencing not only muscle metabolism, thus facilitating muscle fiber conversion, but also the overall energy balance in the body. These findings emphasize the potential of GM modulation as a therapeutic strategy for addressing both sarcopenia and NAFLD. Moreover, dysbiosis can result in increased intestinal permeability, allowing bacterial products to enter the bloodstream and trigger systemic inflammation. This chronic inflammation plays a role in the pathogenesis of NAFLD and sarcopenia, which exacerbates liver injury and fibrosis. In light of these connections, targeting the GM may offer a promising therapeutic intervention for NAFLD and sarcopenia [46]. Hence, the prebiotics and probiotics may have the potential to modulate GM and improve muscle and liver health. Also, the Tarantino et al. study emphasizes that while lactic acid bacteria and bifidobacteria have demonstrated efficacy in preclinical studies, the variability in human populations and the challenges in accurately measuring muscle mass and function prevent the identification of specific probiotics that could be certainly recommended [46].

### 2.2. Prebiotics

Prebiotics are indigestible carbohydrates that can provide a suitable background for the proliferation of beneficial bacteria such as lactobacilli and bifidobacteria [47]. Prebiotics help modulate the GM and prevent liver damage and lipolysis [48]. These carbohydrates improve triacylglycerol concentrations and increase SCFA levels in humans by restoring *Bacteroides* levels [49, 50]. Also, prebiotics in mice improve IR, reduce weight, and strengthen the intestinal barrier [51]. The metabolic effects of prebiotics in humans are associated with conflicting results. Some prebiotics show beneficial results, while others show no difference between groups treated with prebiotics and a placebo-treated group [48]. Prebiotics can show beneficial effects on the nonalcoholic fatty liver by affecting the GM population [52]. These compounds can strengthen the intestinal barrier and reduce metabolic endotoxemia and fat aggregation; therefore, they have the required capability in the prevention and development of obesity and liver disease such as NAFLD [53].

Lactulose is a prebiotic, which can increase the growth of *Bifidobacterium* and *Lactobacillus* and thus improve the symptoms of liver disease [54]. Lactulose treatment of rats with nonalcoholic steatohepatitis showed decreased serum levels of lipopolysaccharides (LPSs) and liver inflammation and promoted the proliferation of bifidobacteria [55]. Fructooligosaccharide and isomaltooligosaccharide are other prebiotics that are effective in preventing and treating NAFLD by decreasing intestinal barrier permeability and IR [56, 57]. In an animal study of mice with NAFLD, the isomaltooligosaccharide prevents weight gain, IR, and metabolic endotoxemia [56]. Oligofructose is a prebiotic that increases *Bifidobacterium* [19, 25, 58]. Also, oligofructose is a prebiotic that can eliminate GM dysbiosis and improve liver histology [58]. Treatment with oligofructose for 8 weeks in seven patients with biopsy-proven NAFLD showed a slight but significant decrease in hepatic aminotransferase (ALT and AST) levels, but according to ultrasound measurements, steatosis was not significantly altered [52].

Inulin-type fructan (ITF) supplementation was first used by Catry et al. This supplement effectively reduces hepatic inflammation by inhibiting nuclear factor kappa-light-chain-enhancer of activated B cells (NF-*κ*B) signaling [59]. ITF therapy appears to be involved in liver health by regulating intestinal microbiota metabolism. In obese women, treatment with ITF had a positive effect on the composition of intestinal microbiota and increased the levels of *Bifidobacterium* and *Faecalibacterium prausnitzii*, which are negatively correlated with serum LPS [60]. One study showed that 8 weeks of ITF usage in rats improves liver histology and reduces portal propionate levels [61]. The aqueous extract of *Ganoderma lucidum* mycelium has the potential as a prebiotic and can provide metabolic health in mice on an HFD. This prebiotic reduces weight and steatosis, improves inflammation and endotoxemia, reduces intestinal barrier permeability, and decreases hepatic toll-like receptor 4 (TLR4) signaling [62].

Probiotic/prebiotic-based therapeutic studies have provided evidence in relieving liver disease. No clinical trials have reported adverse effects in animal models using probiotic or prebiotic treatments. However, prebiotic interventions in humans are few; therefore, to better understand the beneficial effects of prebiotics on NAFLD, more research is required to increase our current knowledge about the effects of these beneficial compounds on liver disease.

### 2.3. Synbiotics

Synbiotics are a combination of prebiotics and probiotics, and the effectiveness of some of them in NAFLD has been evaluated. Most research on the effectiveness of synbiotics focuses on changes in liver enzymes [63]. As a therapeutic agent, synbiotics can prevent or treat NAFLD [64]. Hepatic steatosis and serum LPS in NASH patients were significantly reduced by combination therapy with *Bifidobacterium longum* and fructooligosaccharide [65]. Administration of a synbiotic cocktail consisting of *Bifidobacterium animalis* and inulin for 24 weeks for NAFLD patients significantly reduced hepatic steatosis, which was confirmed by ultrasound, and at the same time, improved liver enzymes were observed in patients [66]. In another combination therapy, using seven probiotics and fructooligosaccharides indicated a greater moderate decrease in hepatic steatosis and fibrosis and lower levels of hepatic aminotransferases (AST and ALT) compared with the placebo group [67]. Fructooligosaccharide with probiotic cocktail including *Lactobacillus rhamnosus*, *Lactobacillus casei*, *Lactobacillus bulgaricus*, *Bifidobacterium longum*, and *Streptococcus thermophilus* was able to improve hepatic stiffness and AST and ALT levels in patients with NASH [68]. One study revealed that although treatment with *Lactobacillus reuteri* with guar gum and inulin was able to reduce hepatic steatosis and weight, no significant reduction was observed in AST and ALT levels [69]. Another study showed that probiotic cocktails and oligofructose improved ALT levels and liver fibrosis (evaluated by elastography) for 28 weeks [70]. The effect of prescribing synbiotics containing *Bifidobacterium animalis* subspecies *lactis* and oligofructose to biopsy-proven NAFLD for 1 year in comparison with placebo on liver fat content (evaluated by MRI) and fibrosis (evaluated by the noninvasive algorithm and transient elastography) has been positive [71]. A meta-analysis study conducted by Ma et al. evaluated the effects of a combination of probiotics such as *Lactobacillus bulgaricus*, *Streptococcus thermophilus*, *Lactobacillus rhamnosus GG*, and *Bifidobacterium longum* with short-chain fructooligosaccharide, which showed a decrease in aminotransferase levels and improve insulin sensitivity [72].

Overall, the results of synbiotic treatment are promising, but the validation of the results in human studies requires the evaluation of effects on subsequent biopsy or NAFLD-related markers. Therefore, further studies are needed to evaluate synbiotics in the regulation of intestinal microbiota and the treatment of liver diseases such as NAFLD.

### 2.4. FMT

FMT is performed to restore and repair GM diversity from a healthy donor to an unhealthy recipient [73]. FMT can help modulate the composition and function of the GM [74] and is known as a new option for NAFLD treatment. FMT from healthy and lean human donors to people with metabolic syndrome significantly improved insulin sensitivity [75]. It has been shown that receiving FMT from healthy nonobese autologous donors to 18 patients with metabolic syndrome reduced IR and the proliferation of SCFA-producing GM after 6 weeks of treatment [76]. Animal studies suggest that FMT affects obesity, IR, and NAFLD [77]. The development of NAFLD has been observed in recipient mice that received FMT from mice with NAFLD [77]. FMT from chow-fed donor mice to HFD mice showed a reduction in triglyceride levels and an improvement in liver histology and liver enzymes (independent of IR). FMT also increases tight junction protein expression such as zonula occludens-1 (ZO-1), thereby reducing intestinal barrier permeability and improving systemic endotoxemia. FMT partially repairs HFD-induced dysbiosis from HFD and increases butyrate concentrations [78]. Significant reductions in fat content and serum transaminase levels were observed in HFD-induced mice and NASH lesions after FMT, indicating a reduction in intrahepatic fat aggregation and proinflammatory cytokines [78]. Microbial gene richness increased in individuals with low microbial gene richness after FMT transmission for 6 weeks, but its beneficial effects were not long-lasting [75]. This suggests that FMT may be performed intermittently for chronic diseases. It is noted that FMT causes a small but significant change in the fecal acetate levels, fecal bile acid reservoir, and downregulation of inflammatory genes in adipose tissue [75, 79, 80]. Therefore, the metabolic characteristics of FMT donors mainly affect insulin sensitivity and bile acid metabolism in the FMT recipient [80].

A study involving 195 human subjects demonstrated that a brief duration of FMT (1 week) was useful and safe [81]. However, human studies have not yet reported the effect of FMT-induced weight loss in people who are overweight or obese [75, 76, 79]. Although FMT can provide more treatments for patients with NAFLD, this possibility still needs more detailed studies [82]. The majority of complications linked to FMT are nonsevere adverse events, predominantly manifesting as transient abdominal pain and changes in intestinal habits. Very rare but severe adverse events have been documented post-FMT, such as sepsis, ileus, bacteremia, perforation, and aspiration [83]. Recently, in a clinical trial in which two adults underwent an FMT, a patient developed *E. coli* bacteremia; unfortunately, this person died because of unintended side effects [84]. Overall, our mechanical understanding of the GM response to FMT is limited, and there are conflicting findings in FMT studies that suggest that more detailed studies are needed in treatments for patients with NAFLD. Nevertheless, FMT treatment is a significant option for patients with liver disease and its related disorders.

### 2.5. Next-Generation Probiotics

At present, due to the rapid evolution of GM, next-generation probiotics are also defined as living microorganisms that, if adequately administered, can be effective in host health [85]. Some of these probiotics, such as *Akkermansia* with a possible role in glucose metabolism or IR, or *Christensenella minuta* and *Parabacteroides goldsteinii*, may have the potential treatment for NAFLD [86–88]. In obese individuals, the abundance of *Christensenella minuta* is lower than in lean individuals in independent cohorts [89]. Treatment with *Christensenella minuta* supplementation at a dose of 10^8^ cells per day for 21 days in mice indicates modification and modulation of the GM, and weight loss was observed [89]. The probiotic *Akkermansia* is involved in the regeneration of the intestinal mucosa and the regulation of intestinal barrier integrity [90]. *Akkermansia* at a dose of 10^10^ bacteria per day over 3 months in obese and Type 2 diabetics (at risk of NAFLD) showed beneficial effects in treated mice, but no beneficial effects on improved insulin sensitivity were observed in either treatment group [91, 92]. However, this probiotic can significantly reduce AST, ALT, and gamma-glutamyl transferase and maintains intestinal barrier integrity [92, 93].

## 3. Future Perspectives

The human microbiome is highly studied in the development of health and disease; it should be noted that the GM is a dynamic, complex, and changeable community. Although several studies have described the effects of GM on animal models and human studies, these studies are not enough and it is unclear whether the microbiome is involved in the pathogenesis of NAFLD. It should be noted that the relationship between microbiome alteration and changes in the immune response in liver disease is not yet well understood, so further studies are required. While some studies have shown promising results, such as improved liver enzymes and reduced intrahepatic triglyceride content, the overall evidence is limited. A systematic review conducted by Tarantino and Finelli notes that the rarity of appropriately powered, randomized, controlled clinical trials involving various centers and populations of different origins makes it difficult to draw definitive conclusions about the therapeutic efficacy of GM modifiers in NAFLD. Furthermore, this systematic review highlights the need for small, careful, and well-controlled studies to investigate the clinical potential of probiotic formulations in affecting markers and pathogenesis of metabolic syndrome [94]. This suggests that while gut microbiome modification holds promise as an emerging therapeutic strategy for NAFLD, more precise research is required to establish its real efficacy. In addition, we need further studies to better understand the complex interactions between GM with other members of the microbial community or fungal species with the host. Manipulation of GM by FMT can help us better know their function and describe new horizons for disease prevention and treatment. In general, the day-to-day progress of knowledge about GM helps to develop diagnostic tools, prognosis, and treatments based on GM modification to manage liver disease. Currently, research should focus on identifying microorganisms that are known to be indicators of liver damage and contribute to disease progression. It can be acknowledged that intestinal microbiota dysbiosis is a suitable noninvasive diagnostic marker for liver disease because the composition and structure of the microbial community in different diseases are unique.

## 4. Conclusion

Following the prevalence of obesity, NAFLD is on the rise worldwide. It suggests that NAFLD is closely associated with metabolic diseases. GM is thought to be involved in the pathophysiology of NAFLD because obesity can affect the function and composition of the GM community by reducing microbial gene richness and leading to metabolic changes, which can have negative consequences for the health of the host. At the moment, most therapeutic interventions focus on changing the GM, which in combination with lifestyle modification can have promising results. Modification of GM through specific diet patterns, probiotics/prebiotics/synbiotics, and FMT has yielded promising results. Among the strategies mentioned, probiotics are frequently evaluated in animal and human studies, and the results of studies show their positive effects on NAFLD, although long-term studies can validate their beneficial effects on various liver diseases. In general, modification of the GM can help restore health to people with NAFLD. Animal studies have partially revealed the role of GM in host health, but solving this puzzle requires comprehensive studies in large human groups that can help gather valuable data and improve our current information about GM in NAFLD treatment.

## Data Availability

The authors have nothing to report.
